# Moral reasoning in women with posttraumatic stress disorder related to childhood abuse

**DOI:** 10.3402/ejpt.v7.31028

**Published:** 2016-11-08

**Authors:** Anthony Nazarov, Victoria Walaszczyk, Paul Frewen, Carolina Oremus, Ruth Lanius, Margaret C. McKinnon

**Affiliations:** 1 Department of Psychiatry and Behavioural Neurosciences, McMaster University, Hamilton, ON, Canada; 2 Mood Disorders Program, St. Joseph's Healthcare Hamilton, Hamilton, ON, Canada; 3 Department of Psychiatry, Western University, London, ON, Canada; 4 Department of Psychology, Western University, London, ON, Canada; 5 Department of Neuroscience, Western University, London, ON, Canada; 6 Homewood Research Institute, Guelph, ON, Canada; 7 Imaging Division, Lawson Health Research Institute, London, ON, Canada

**Keywords:** Morals, social perception, stress disorders, posttraumatic, adult survivors of child abuse, moral judgment

## Abstract

**Background:**

Preliminary evidence suggests that relative to healthy controls, patients with posttraumatic stress disorder (PTSD) show deficits on several inter-related social cognitive tasks, including theory of mind, and emotion comprehension. Systematic investigations examining other aspects of social cognition, including moral reasoning, have not been conducted in PTSD stemming from childhood trauma.

**Objective:**

To conduct a comprehensive assessment of moral reasoning performance in individuals with PTSD stemming from childhood abuse.

**Method:**

Moral reasoning performance was assessed in 28 women with PTSD related to prolonged childhood trauma and 19 matched healthy controls. Performance was assessed using 12 modified moral dilemmas and was queried in three domains: utilitarian/deontological sacrificial dilemmas (personal and impersonal), social order vs. compassion, and altruism vs. self-interest. Participants were asked whether a proposed action was morally acceptable or unacceptable and whether or not they would perform this action under the circumstances described.

**Results:**

Women with PTSD were less likely to carry out utilitarian actions in personal, sacrificial moral dilemmas, a choice driven primarily by consequential intrapersonal disapproval. Increased concern regarding intrapersonal disapproval was related to higher symptoms of guilt in the PTSD group. Patients with PTSD demonstrated less altruistic moral reasoning, primarily associated with decreased empathic role-taking for beneficiaries.

**Conclusions:**

Women with PTSD due to childhood trauma show alterations in moral reasoning marked by decreased utilitarian judgment and decreased altruism. Childhood trauma may continue to impact moral choices made into adulthood.

**Highlights of the article:**

- Moral judgment processing was found to be altered in women with PTSD related to chronic childhood trauma.
- In comparison to healthy women, women with PTSD were less likely to approve utilitarian actions when required to assume their agency in dilemmas involving the infliction of direct physical harm.
- Decreased likelihood of utilitarian action approval by the PTSD sample was driven by significantly enhanced endorsement of guilt and shame as consequences of such actions.
- Endorsement of guilt and shame in moral dilemmas was related to increased severity of current clinical symptoms of guilt.

Posttraumatic stress disorder (PTSD) is a debilitating mental illness that may develop after exposure to traumatic or psychologically stressful life events and is marked by symptoms of re-experiencing, avoidance, negative cognitions, and arousal alterations. Individuals with PTSD often display alterations in intrapersonal function across multiple domains, including work, intimate relationships, and familial interactions (Cloitre, Miranda, Stovall-McClough, & Han, 2005; DiLillo, 2001; Evans, McHugh, Hopwood, & Watt, 2003). Given the critical role of social support in the recovery process for PTSD (Charuvastra & Cloitre, 2008), a decreased capacity to interact optimally with others represents a critical target for treatment intervention. Here, we explore moral reasoning performance among individuals with PTSD stemming from developmental trauma exposure, with a particular emphasis on differences in motivation for moral choices among this sample. Given previous research suggesting that exposure to developmental trauma alters key socio-cognitive processes that unfold over critical developmental periods (e.g., theory of mind and emotion comprehension; Saxe, Carey, & Kanwisher, 2004; Wellman, Cross, & Watson, 2001; Aguert, Laval, Lacroix, Gil, & Le Bigot, 2013), we predicted that exposure to trauma in childhood would have long-standing effects on moral reasoning performance that persist into adulthood.

The ability to interact with the outside world is heavily dependent on early-life experiences and environmental feedback. Socio-cognitive skills develop over the first 5 years of life and are honed into adolescence (Blakemore & Choudhury, 2006; Wellman et al., 2001). Accordingly, this developmental period is associated with inherent risks and opportunities, where childhood experiences may shape long-lasting patterns of behavior, including social cognition. In cases of developmental trauma, particularly that which is chronic and inflicted by trusted individuals who would be expected to provide safety and support, children may develop distinct behavioral responses that include dissociation and learned helplessness (Lanius, Frewen, Vermetten, & Yehuda, 2010; Lanius, Vermetten, et al., 2010). These responses may represent optimal adaptations for survival in environments where the option of escape is persistently non-existent (Lanius, Frewen, et al., 2010) and that differ qualitatively from symptoms experienced in PTSD stemming from a single-blow trauma or trauma experienced during adulthood (Lanius, Vermetten, et al., 2010).

Critically, the unique cognitive, emotional, and behavioral profiles (e.g., dissociation) that protect an individual during early-life adversity may be incongruent with safe environments encountered during adulthood and may contribute to the interpersonal dysfunction and functional impairment frequently observed in adult survivors of developmental trauma (Cloitre et al., 2005; Lanius, Bluhm, & Frewen 2011). Here, alterations in social cognitive functioning (e.g., ability to recognize emotion, empathic responding, perspective-taking) stemming from childhood experience would be expected to contribute significantly to interpersonal disruptions observed in adulthood. For example, the ability to engage in moral reasoning unfolds over a lengthy developmental window and is highly dependent on the emergence of moral emotions, the maturation of empathic and perspective taking abilities, and optimal attachment styles (Malti, Ongley, Killen, & Smetana, 2014). Alterations in these processes as the result of childhood trauma exposure would be expected to result in long-standing differences in moral reasoning performance relative to that of individuals that did not experience developmental trauma exposure, with differences persisting into adulthood (Fish-Murray, Koby, & Van der Kolk, 1987).

Indeed, accumulating evidence suggests that social cognition, the ability to interact optimally and to navigate the social world, may be altered in adults exposed to psychological trauma. Previous studies, including work conducted by our laboratory, have shown alterations in empathic responding (Nietlisbach, Maercker, Rössler, & Haker, 2010; Parlar et al., 2014), recognition of speech prosody (Nazarov, Frewen, et al., 2015), theory of mind (Mazza et al., 2012; Nazarov et al., 2014), and direct gaze processing (Steuwe et al., 2015) in this population. A reoccurring theme surrounding alterations in social cognition among individuals with PTSD is alterations in its performance in emotionally salient social contexts. For example, Nazarov et al. (2014) reported that individuals with PTSD are slower to identify complex mental states for emotionally salient trials only but not neutral trials. In a related study exploring self-reported empathic concern in women with PTSD, Parlar et al. (2014) found increased personal distress in response to emotionally charged social situations. Moral reasoning is a highly complex domain of social cognition that draws upon social norms to frame human interactions, requiring the engagement of theory of mind, self-referential processing, and empathy, and is seldom void of emotional salience (Greene & Haidt, 2002; Northoff & Bermpohl, 2004; Reniers et al., 2012). Accordingly, we sought to explore moral reasoning performance in women with PTSD due to childhood trauma, where exposure to trauma during critical developmental periods would be expected to alter the development and expression of this key component of social interaction.

Although the study of morality has been left mainly to philosophy, recent advances in developmental (Turiel, 2008; Walker, 1989), forensic (Blair, 2007), and neurocognitive psychology (Greene, Sommerville, Nystrom, Darley, & Cohen, 2001) have greatly increased our understanding of how our minds deliberate moral issues, and in turn, direct moral actions. Indeed, research surrounding moral cognition is expanding rapidly, with several frameworks [e.g., dual-process theory (Greene et al., 2001; Greene, Nystrom, Engell, Darley, & Cohen, 2004); event–feature–emotion complex (EFEC; Moll, Zahn, De Oliveira-Souza, Krueger, & Grafman, 2005)] being recently proposed (see an extensive review of moral cognition frameworks by Moll et al., 2005). There are two contrasting aspects to the classic understanding of morality—utilitarianism and deontology. Utilitarianism involves the understanding that the correct action is the one that results in greater good, regardless of the means to an end (Mill, 1998). By contrast, deontology posits that certain actions are always amoral, regardless of how good the intentions or outcomes are (Kant, 1959).

The dual-process theory proposed by Greene et al. (2001, 2004) provides one framework for the complex process of moral decision-making, particularly in situations where deontological and utilitarian values are in conflict. Here, it is generally understood that whereas more utilitarian choices are based upon effortful cognitive reasoning, deontological choices draw upon more innate, emotional responses. Greene et al. (2001, 2004) suggest further that there are two types of moral dilemmas, personal and impersonal. A personal moral dilemma places the participant in a situation where he/she must decide whether or not to inflict harm directly onto another person. An example of this type of dilemma is the footbridge dilemma (Thomson, 1986)—you must throw someone onto the tracks of an oncoming out-of-control trolley that is imminently going to kill five people. The body of the victim that you pushed will stop the trolley and consequently save the five people. In an impersonal moral dilemma, the harm to the victim occurs less directly. For example, the modified trolley dilemma (Thomson, 1986) also involves saving the lives of five people by killing one, except here the path of the trolley is redirected by means of a switch. Personal moral dilemmas tend to evoke a stronger emotional response, making the decision more complex and harder to resolve (Greene et al., 2004). This increased complexity is thought to arise due to a conflict between the “emotional” response (avoidance of inflicting direct harm) and the purely “cognitive” response (saving five lives over one despite the negative emotional connotations) (Greene et al., 2004).

Critically, moral reasoning relies heavily on a network of neural regions shown previously to be impacted in PTSD including the orbitofrontal cortex (OFC) (Beer, Heerey, Keltner, Scabini, & Knight, 2003; Moll & de Oliveira-Souza, 2007a), dorsolateral prefrontal cortex (DLPFC) (Greene et al., 2004; Moll & de Oliveira-Souza, 2007a), anterior cingulate cortex (ACC) (Greene et al., 2004), and amygdala (Berthoz, Grèzes, Armony, Passingham, & Dolan, 2006). In parallel, an expanding body of evidence indicates that patients with PTSD demonstrate structural and/or functional changes in the prefrontal cortex, ACC, OFC, and amygdala (see Francati, Vermetten, & Bremner, 2007; Liberzon & Sripada, 2008 for review), with deficits emerging in fronto-temporally mediated domains of cognition, including working memory and attention required for performance of social reasoning tasks (McKinnon & Moscovitch, 2007). Greene et al. (2001) suggest that whereas the OFC is responsible for eliciting emotional responses, the DLPFC is involved in evoking a cognitive response. When both of the responses are strong, this conflict may be resolved by the ACC. Notably, the dual-process model has been the subject of recent criticism (Moll & De Oliveira-Souza, 2007a, 2007b), where, for example, patients with ventromedial prefrontal cortex damage displayed increased emotional responses during the Ultimatum Game (Koenigs & Tranel, 2007)—a result in direct contrast to model predictions. By contrast, the recent EFEC model suggests that moral cognition arises from the integration and interaction of context-dependent knowledge, social perception and comprehension, and emotional motivation (Moll et al., 2005). Other alternative models include the moral sensitivity hypothesis and the structured–event–complex framework; each is discussed in detail in a topical review (see moral cognition frameworks by Moll et al., 2005). Despite the lack of consensus on a unified theory of moral cognition, there is an urgent need to explore moral reasoning performance in patients with PTSD, given the potential contribution of disruptions in moral reasoning performance to poor interpersonal functioning in this population.

To obtain a comprehensive assessment of moral reasoning performance in the present study, we examined performance on moral dilemmas exploring three different domains: utilitarianism vs. deontology, social order vs. compassion, and altruism vs. self-interest. Participants were asked whether a proposed action was morally acceptable or unacceptable and whether or not they would perform this action under the circumstances described. We hypothesized that individuals with PTSD due to chronic childhood trauma would experience alterations in moral reasoning performance both as a function of early life experience and ongoing alterations in the cognitive and emotional processes impacted in PTSD.

## Methods

### Participants

Forty-seven women were recruited to participate in this study; 28 women with a primary diagnosis of current PTSD related to childhood abuse (PTSD group; mean age 42.0 [SD=11.6] years) and 19 female healthy controls of similar age (HC group; mean age 36.1 [SD=13.5] years). Women with PTSD were recruited at the London Health Sciences Centre (LHSC; London, Ontario, Canada) through outpatient programs. The HC subjects were recruited through word of mouth and local advertisements at LHSC and St. Joseph's Healthcare Hamilton (Hamilton, Ontario, Canada). HC participants had no current or lifetime history of psychiatric illness.

Diagnosis of PTSD was confirmed via the Structured Clinical Interview for DSM-IV (SCID) (First, Spitzer, Gibbon, & Williams, 2002). PTSD symptom severity was assessed using the Clinician-Administered PTSD Scale (CAPS) (Blake et al., 1995), and depression symptom severity was measured with the Beck Depression Inventory (BDI) (Beck, Steer, & Brown, 1996). Symptoms of dissociation and childhood trauma history were assessed by the Multiscale Dissociation Inventory (MDI) (Briere, 2002) and the Childhood Trauma Questionnaire (CTQ) (Bernstein et al., 1994), respectively. Demographic and clinical summaries are provided in Table 1. Healthy controls were administered the same measures in order to rule out the presence of current and past psychiatric illness (using SCID) and history of childhood maltreatment (using the CTQ). Exclusion criteria for all groups were: (1) substance-use related disorder within the past 6 months as determined by the SCID; (2) use of alcohol or illicit psychoactive substance within 48 h of testing; (3) significant medical illness; (4) history of head injury with loss of consciousness lasting more than 60 s; and (5) history of neurological disease. The study sample was drawn from the same pool of participants described in Nazarov et al. (2014).

**Table 1:** Clinical and demographic characteristics of study sample

Characteristic	Control (*n*=19)	PTSD (*n*=28)
	*n*	*N*
Sex		
Male	0	0
Female	19	28
	Mean	Mean
Age	36.1 (13.5)	42.0 (11.6)
Education	16.3 (2.4)	13.8 (2.4)*
CAPS	0.1 (0.5)	79.4 (16.2)*
BDI	2.5 (4.4)	30.8 (12.4)*
Childhood Trauma Questionnaire		
Emotional abuse	5.9 (2.14)	18.5 (5.2)*
Physical abuse	5.5 (1.1)	13.0 (5.7)*
Sexual abuse	5.2 (0.4)	15.5 (7.3)*
Emotional neglect	7.4 (2.2)	17.9 (4.9)*
Physical neglect	6.2 (1.7)	11.6 (5.5)*
MDI (Total)	34.7 (6.0)	75.1 (21.9)*
Disengagement	7.6 (2.4)	17.0 (4.0)*
Depersonalization	5.2 (0.4)	10.8 (5.1)*
Derealization	5.5 (1.7)	11.7 (4.2)*
Emotional constriction	5.5 (1.1)	13.0 (6.1)*
Memory disturbance	5.6 (1.5)	12.1 (4.5)*
Identity dissociation	5.2 (0.5)	10.6 (6.2)*

Values are *n* or mean (standard deviation). BDI: Beck Depression Inventory; CAPS: Clinician-Administered PTSD Scale; MDI: Multiscale Dissociation Inventory; PTSD: posttraumatic stress disorder.

* Significant group effect (*p*<0.05).

### Moral judgment task

This task was designed to test participants’ on-line ability to reason about complex moral situations and represents a modified series of dilemmas based on Greene et al. (2001). A total of 12 moral dilemmas were presented individually (see Appendix A for a complete list of moral dilemmas). Each dilemma and response options were read aloud by the interviewer, with a written copy of the dilemma being available to the participant. Four variables were recorded for each dilemma: moral knowledge decision (“morally okay or not okay”), moral knowledge justification (“why is it morally okay or not okay?”), moral intent decision (“would you do it?”), and moral intent justification (“why or why not would you do it?”). To reduce memory demands, our stories were relatively brief (50–75 words) with both stories and questions available for inspection until a response was made. Responses were audio-recorded and transcribed. The moral dilemmas were equally divided by type: six dilemmas where the actions involved physical harm and six dilemmas where the actions involved no physical harm. The six physical harm dilemmas were further categorized into three personal (direct infliction of harm) and three impersonal (indirect infliction of harm) dilemmas. The physical harm moral dilemmas were of primary focus in this investigation as they elicited the dual cognitive and emotional processes. The non-physical harm dilemmas contained four moral dilemmas probing social order vs. compassion, and two moral dilemmas probing altruism vs. self-interest. Two blind, independent raters qualitatively categorized the judgments behind moral decisions using the moral judgment categorization found in Gibbs, Basinger, and Fuller (1992) as a guideline (see Appendix B for categories scored in this sample). Conflicting categorizations were resolved upon rater consensus.

### Statistical methods

To examine group differences on the demographic and clinical variables, two-tailed independent-samples *t-*tests were used. All analyses were preceded by the Shapiro–Wilk test of normality and Levene's test of heteroskedasticity. Group differences in moral decision-making were analyzed using a mixed-design ANOVA, with diagnosis as a between-subjects factor and physical harm type (physical harm/no physical harm), harm infliction type (personal/impersonal), or moral knowledge vs. intent as a repeated measure. Associations were calculated using Pearson's *r* or Spearman's *r*_s_ (two-tailed; α=0.05). Effect sizes were estimated by partial eta-squared ($\eta_{p}^{2}$) and Cohen's *d*. Fisher's exact test and odds ratios (OR) were used to compare group differences in qualitative responding. Analyses were conducted with SPSS 21 and R (3.0) statistical software. Qualitative scoring was conducted with QSR NVivo 10.

## Results

We found that one out of four variables analyzed in the repeated-measures ANOVAs marginally failed the Levene's test of equality of error variance, highlighting a potential issue with heteroskedasticity. However, the Box's Test of Equality of Covariance Matrices passed for these analyses. In light of equal covariance matrices and only one variable marginally failing the Levene's test, we, therefore, proceeded with using repeated-measures ANOVA for our quantitative analyses.

### Clinical and demographic characteristics

There were no statistically significant group differences in age; however, women with PTSD had significantly fewer years of education than controls (*p*=0.001; see Table 1 for demographic and clinical characteristics). As expected, patients with PTSD had significantly higher scores on the CAPS, BDI, CTQ, and MDI compared with controls (*p*'s<0.05).

### High-conflict physical harm dilemmas

#### Quantitative analysis

**Fig. 1:**
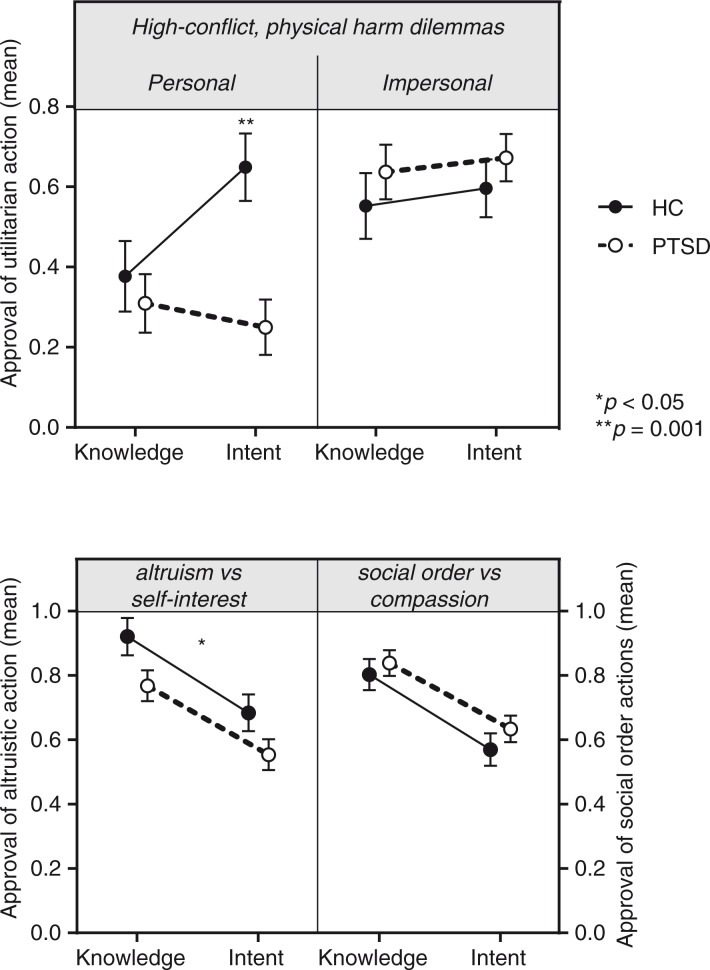
Mean approval rates of moral dilemmas in patients with PTSD compared to HC.

For moral choices involving high-conflict physical harm dilemmas, there was a significant interaction between morality type (knowledge/intent), harm type (personal/impersonal), and PTSD diagnosis based on a repeated-measures ANOVA (*F*(1, 45)=7.36, *p*=0.009, $\eta_{p}^{2}$=0.141; Fig. 1; N.B. one out of the four independent variables in this ANOVA was heteroskedastic). *Post-hoc* testing revealed that compared to healthy controls, patients with PTSD were less likely to approve of a utilitarian action only in situations where physical harm was to be personally inflicted (*t*(45)=3.67, *p*=0.001). However, there were no significant differences between patients and controls on impersonal physical harm dilemmas (*F*(1, 45)=0.014, *p*=0.9; knowledge: *t*(45)=0.79, *p*=0.43; intent: *t*(45)=0.82, *p*=0.42).

#### Qualitative analysis

**Fig. 2:**
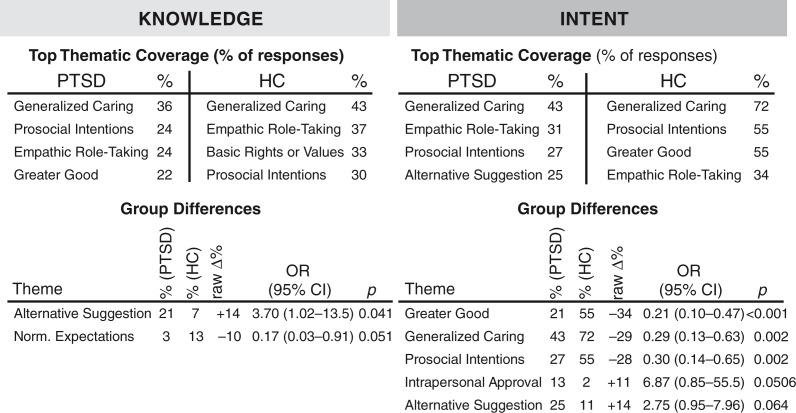
Most common themes and group differences found in personal moral dilemmas.

Although patients with PTSD and HCs endorsed comparable rates of moral approval (moral knowledge) for utilitarian action involving high-conflict personal dilemmas, several qualitative differences emerged between groups (see Fig. 2 for most common themes and group differences). Specifically, patients with PTSD were more likely to avoid the dichotomy of the utilitarian/deontological trade-off and suggest instead the possibility of an alternative outcome that avoided the need to execute the utilitarian action in order to save others (OR=3.70, 95% confidence interval, CI [1.02–13.50], *p*=0.041). There was also a trend toward differences in expression of normative expectations across groups on moral knowledge of personal harm dilemmas. Patients with PTSD were less likely to mention normative expectations (e.g., “people deserve to live,” “it's the human thing to do,” “how could anyone do/not do this?”) in comparison to controls (OR=0.17, 95% CI [0.03–0.91], *p*=0.051).

Judgments surrounding decisions to *personally* carry out the utilitarian actions in high-conflict personal harm moral dilemmas varied greatly between PTSD and HC samples. PTSD patients were significantly less likely to mention the greater good (OR=0.21, 95% CI [0.10–0.47], *p*<0.001), generalized caring (OR=0.29, 95% CI [0.13–0.63], *p*=0.002), and prosocial intentions (OR=0.30, 95% CI [0.14–0.65], *p*=0.002) when inquired what choice they would make if presented with such situations. Instead, PTSD patients were more likely to mention concerns about intrapersonal approval (e.g., guilt, shame) (OR=6.87, 95% CI [0.85–55.5], *p*=0.051) and alternative suggestions (OR=2.75, 95% CI [0.95–7.96], *p*=0.064) in comparison to HC (trending significance) (see Table 2 for example quotes from the PTSD groups). Indeed, all individuals mentioning intrapersonal approval and alternative suggestions refused to carry out the utilitarian action if personally presented with the dilemmas.

**Table 2:** Selected responses made by patients with PTSD when contemplating own agency (moral intent) in personal (high-conflict) sacrificial moral dilemmas

1. It would haunt me for as long as I would live.
2. I would probably shoot myself. It would be too difficult to shoot and kill my crew member.
3. I would try other tactics first.
4. If they all stay together then no one is going to feel guilty that they killed someone.
5. I wouldn't want to shoot him and no one should have to carry that act with them forever.
6. I would suggest another method of saving everyone.
7. I would not do it based on my own inability to cope with the guilt and it may be greater knowing that it cost people their lives.
8. I could not live with myself after killing another person.
9. I would dissociate. I wouldn't be able to deal with it and would have to get someone else to do it. I'd be freaking out and want to hurt myself instead.
10. I would throw myself over the boat. Will not be able to live if I cut a woman's throat.

### Non-physical harm dilemmas

#### Quantitative analysis

For non-physical harm dilemmas, there was a significant interaction between morality domain (altruism/social order) and PTSD diagnosis based on a repeated-measures ANOVA (*F*(1, 45)=4.48, *p*=0.040, $\eta_{p}^{2}$=0.09; Fig. 1; N.B. one out of the four independent variables in this ANOVA was heteroskedastic). In comparison to patients with PTSD, HCs were more likely to approve and carry out altruistic actions when pitted against self-interest (*F*(1, 45)=5.55, *p*=0.023, $\eta_{p}^{2}$=0.11; Fig. 1).

#### Qualitative analysis

In describing their reasoning behind the moral permissibility of an altruistic act, patients with PTSD were more likely than HCs to mention personal freedoms (OR=14.1, 95% CI [0.78–252], *p*=0.019) and were less likely to mention generalized caring (OR=0.38, 95% CI [0.16–0.90], *p*=0.034). When considering their agency in the dilemma, patients with PTSD were less likely than HCs to assume an empathic stance toward the potential beneficiary of the altruistic act (OR=0.33, 95% CI [0.12–0.92], *p*=0.043).

There were no significant differences between patients and controls on moral dilemmas probing social order vs. compassion.

### Relation to clinical symptoms and demographic variables

Within the patient sample, individuals who have mentioned intrapersonal approval as central to the reasoning surrounding their moral choices reported significantly higher symptoms of guilt due to omission/commission on the CAPS than did those who did not (*t*(14)=2.47, *p*=0.027). There were no differences, however, in PTSD severity (total CAPS score), depressive symptoms (BDI), and dissociative symptoms (MDI total score) between these groups (all ∣*t*∣<1.0; *p*>0.3).

We found no significant correlations between age and moral decision (moral knowledge or moral intent) for each moral dilemma style (physical harm—personal, physical harm—impersonal, social order vs. compassion, and altruism vs. self-interest) in both patients (all *p*'s>0.1; ∣*r, r*_*s*_∣<0.31) and HC (*p*'s>0.25; ∣*r, r*_*s*_∣<0.28). Similarly, we found no significant correlations between years of education and moral decision, analyzed separately for patients with PTSD and HC (all *p*'s>0.17; ∣*r, r*_*s*_∣<0.27). Dilemma choices were not associated with our primary PTSD severity index (CAPS), symptoms of depression (BDI), or dissociation (MDI; all *p*'s>0.14; ∣*r, r*_*s*_∣<0.28).

## Discussion

To our knowledge, this is the first study to explore moral decision-making performance in PTSD due to childhood trauma. Here, we used a novel study design to discern both quantitative and qualitative differences in moral reasoning performance, allowing us to explore different types of moral dilemmas and the potential divergence between moral reasoning and moral intent. When presented with high-conflict personal moral dilemmas, in comparison with healthy women, women with PTSD were less likely to mention the benefit to greater good and were more likely to mention intrapersonal approval/disapproval (guilt/shame) as a consequence of carrying out utilitarian actions. Subsequently, women with PTSD described themselves as less likely to carry out a utilitarian action despite making similar decisions to controls concerning whether the action was morally acceptable or not acceptable. Interestingly, concerns regarding intrapersonal approval surrounding moral judgments were related to the presence of associated clinical symptoms of guilt surrounding perceived acts of omission and commission. Notably, however, moral reasoning performance was not associated with other clinical features, including dissociation, or severity of PTSD or depressive symptoms. Finally, when presented with moral dilemmas exploring altruism vs. self-interest decisions, in comparison to healthy women, women with PTSD were less likely to endorse and carry out an altruistic action.

In this study, we presented participants with a range of moral dilemmas that queried moral judgment on topics that included altruism vs. self-interest, law vs. compassion, and sacrifice for the greater good (deontology vs. utilitarianism). Critically, our qualitative analysis revealed that although women with PTSD and HCs made similar judgments about the moral permissibility of a utilitarian action that required each group to exert direct harm, women with PTSD were less likely to endorse personally carrying out those actions if hypothetically required to do so. This analysis also revealed that primarily among the PTSD group only, feelings of guilt and shame were cited as reasons to not follow through with these sacrificial actions. By contrast, healthy subjects were more likely to carry out hypothetical utilitarian actions requiring direct harm despite endorsing them as morally unacceptable.

### Role of guilt and shame

Guilt and shame are moral emotions that are expressed when one's behavior does not align with social and/or personal moral standards (Tangney, Stuewig, & Mashek, 2007) thus serving as an adaptive moral compass that utilizes emotional processing based on existing sociocognitive schemas. Recent theories postulate that these experiences of shame and guilt may play a central role in PTSD symptomology (Herman, 2011; Leskela, Dieperink, & Thuras, 2002; Matos & Pinto-Gouveia, 2010). For example, guilt and shame symptoms are frequently associated with perceived perpetration of and exposure to moral transgressions in military members with PTSD, potentially mediating the relation between trauma exposure and symptom severity in PTSD and major depressive disorder (MDD) (see Nazarov, Jetly, et al. 2015, for review). Symptoms of shame and guilt also arise frequently in individuals who have been victimized (e.g., through physical and sexual assault, transportation accidents, and developmental trauma; Budden, 2009; Herman, 1992). Here, assuming unwarranted blame for traumatic events over which one had no control may alter one's sense of self, resulting in feelings of alienation and decreased access to social support (Litz et al., 2009). These maladaptive symptoms of guilt and shame, particularly following victimization, impede recovery.

In our sample, we found that clinical symptoms of guilt (DSM-IV nomenclature) were associated with the propensity to endorse guilt and shame as consequences of utilitarian actions when required to undertake a morally ambiguous action that involved one's agency. Here, clinical symptoms of guilt and shame in our patient sample may have exerted a priming effect on moral decision-making during reasoning about the hypothetical dilemmas. Indeed, previous research has shown that inducing feelings of guilt alters moral reasoning in healthy populations (De Hooge, Zeelenberg, & Breugelmans, 2007). We speculate that individuals with PTSD may experience increased awareness of the debilitating consequences of guilt and shame due to the nature of their symptomatological profile and history of past symptoms of guilt. As a result, they may be more accurate at predicting how these moral emotions will impact intrapersonal approval following the morally transgressive actions.

Notably, our findings of decreased utilitarian judgment and increased endorsement of moral emotions among the PTSD group align well with the EFEC model (Moll et al., 2005; Moll, De Oliveira-Souza, & Zahn, 2008), which would predict decreased utilitarian judgments among individuals experiencing higher symptoms of guilt. Among individuals with PTSD, a significant body of evidence points further to differing patterns of neural activation among individuals who exhibit symptoms of hyperarousal or re-experiencing reactivity compared to those who experience dissociative symptomatology (Bremner, 1999; Lanius, Bluhm, Lanius, & Pain, 2006; Lanius, Brand, Vermetten, Frewen, & Spiegel, 2012; Lanius et al., 2002; Lanius, Vermetten, et al., 2010). Here, functional neuroimaging studies indicate that individuals who report re-experiencing a traumatic memory in response to script provocation with concomitant psychophysiological hyperarousal exhibit reduced activation in the medial prefrontal and rostral ACC, accompanied by increased amygdala reactivity. Fronto-limbic suppression models of emotions would explain these reliving responses as mediated by a failure of prefrontal inhibition or top-down control of limbic regions. Applying the EFEC model to the symptoms of re-experiencing, Moll et al. (2008) predict that the altered experience of emotions may be based on the impaired integration of context-dependent social knowledge represented in the ventromedial frontal cortex. By contrast, individuals who also report symptoms of depersonalization and derealization (with concomitant psychophysiological hypoarousal) show increased activation in the rostral ACC and the medial prefrontal cortex during states of depersonalization/derealization, suggesting that the latter responses are mediated by midline prefrontal inhibition of the limbic regions (Frewen and Lanius, 2006; Lanius, Frewen et al., 2010; Lanius et al., 2012).

Critically, these differential patterns of activation, relating to behavioral subtypes of PTSD, may result in contrasting patterns of moral reasoning performance where patients with a primarily state hyperarousal response may show moral reasoning performance somewhat similar to that of patients with ventromedial lesions, reflecting a failure of prefrontal inhibition over emotional responses engendered by provocation stimuli (e.g., moral dilemmas). By contrast, patients with a primarily state hypoarousal response may show a contrasting pattern, reflecting prefrontal inhibition of emotional response and concomitant hypoemotionality. These patterns remain to be verified in future studies. It will also be critical to examine behaviorally the effects of different dissociative states on moral reasoning performance (Frewen & Lanius, 2015).

It is well established that knowledge of moral rules does not necessarily translate to actions that follow the same principles, with psychopathy being an extreme example where a divide exists between moral knowledge and actual behavior (Cima, Tonnaer, & Hauser, 2010). Studies investigating psychopathy have alluded to the reduction in emotional processing of guilt that may be antecedent to moral behavior (Blair et al., 1995). Interestingly, in our study, when queried whether an action is morally acceptable or not, minimal endorsements of guilt and shame were made in both the PTSD and HC groups. Requiring participants to reason as to whether or not they would undertake the morally ambiguous action (despite potential moral objections) may be a more representative indicator of moral reasoning ability, as it may heighten the emotional salience of the dilemma. Indeed, some paradigms of moral reasoning implement techniques (e.g., closing of eyes during scene descriptions) that have been shown to maximize perspective-taking and by doing so may evoke emotional processing that may be naturally lacking in laboratory-based artificial settings and hypothetical scenarios (Amit & Greene, 2012; Caruso & Gino, 2011). In the present study, we attempted to bridge the divide between moral knowledge and moral action by requiring the participants to assume the role of the agent.

### Role of perspective-taking and empathy

Given similar judgments of the acceptability of utilitarian actions among women with PTSD and controls, it is possible that women with PTSD experienced a comparable ability to assume the often conflicting (varying with values, actions, intentions, feelings) perspective of characters depicted in the dilemmas. Indeed, Nazarov et al. (2014) found that individuals with PTSD due to developmental trauma showed decreased theory of mind performance only in situations relating to familial interactions. Critically, none of the dilemmas presented in this study required the participants to assume a familial role in relation to the characters in the dilemmas. Surprisingly, some research points to improvements in perspective-taking when feeling guilty (Yang, Yang, & Chiou, 2010). In an affect induction study of health participants, Yang et al., (2010) found that subjects induced to experience feelings of guilt demonstrated improved perspective-taking performance over the neutral state condition. In contrast, individuals induced to experience shame showed a reduction in perspective-taking. Future studies exploring the interplay of shame, guilt, theory of mind, and moral judgment are warranted.

The dual-processing theory of moral judgment follows the premise that moral judgment is the product of the interplay between cognitive and (at times conflicting) emotional processing (Greene, Morelli, Lowenberg, Nystrom, & Cohen, 2008; Paxton & Greene, 2010). In our sample, healthy women were more likely to override the emotional attributes of high-conflict dilemmas and instead emphasize the utilitarian outcomes behind their endorsement of utilitarian action. This difference may relate, in part, to differences in empathic responding between individuals with PTSD and HCs (Nietlisbach et al., 2010; Parlar et al., 2014). Research on empathy and its relation to moral judgment has been scarce. Patil and Silani (2014) reported an association between increased utilitarian judgment and decreased empathic concern in individuals with trait alexithymia. Given previous findings of decreased empathic concern in PTSD (but increased personal distress in relation to others’ difficulties) (Parlar et al., 2014), we might have expected increased approval of utilitarian actions in our PTSD sample compared to controls. In contrast, our results indicate that despite alterations in empathic concern alterations among individuals with PTSD, utilitarian thought was significantly lower in our PTSD sample when compared with healthy subjects. One line of reasoning that may aid in interpreting these contradictory findings is the PTSD group's reasoning behind the decreased tendency for utilitarian thought. Individuals with PTSD focused on interpersonal disapproval as opposed to the emphasis on the violation of basic rights of the victim reflecting a preoccupation with their internal emotional states. Indeed, Parlar et al. (2014) and Nietlisbach et al. (2010) found increased personal distress in response to emotional social contexts in individuals with PTSD. The increase in personal distress in response to other's suffering may represent an erosion of the boundary between self and other, thus heightening the salience of personal consequences of a morally objectionable act. Interestingly, women with PTSD were more likely to generate alternative suggestions in lieu of choosing a utilitarian option. Personal discomfort and fear of emotionally charged contexts may further prevent an individual from undertaking a difficult moral decision, instead leading to inaction (in this case the avoidance of utilitarian action). Future studies should disentangle the differences between distress-related inaction and deliberate deontological choice.

### Altruism

In our study, patients with PTSD were less likely to endorse and carry out altruistic behaviors in comparison with HC. Altruism is a prosocial behavior that is driven by concern for others rather than concern for oneself and is accompanied with inherent personal costs (e.g., personal risk during the action or as an outcome, opportunity cost) (Eisenberg, 2014). Empathy and perspective-taking are socio-cognitive processes that are central to evoking altruistic behaviors (Eisenberg, Eggum, & Di Giunta, 2010), and rely on common neural circuitry (FeldmanHall, Dalgleish, Evans, & Mobbs, 2015). Interestingly, individuals high in altruism engage in neural networks related to empathy and theory of mind more so than individuals who are less altruistic (Haas et al., 2015). FeldmanHall et al. (2015) reported that altruistic behavior is predicted by increased empathic concern for others but not by levels of personal distress. Previous research examining the relation between early childhood trauma and empathic abilities support our observation of reduced altruistic behaviors in patients with PTSD. As demonstrated by Parlar et al. (2014), women with PTSD exposed to chronic childhood trauma displayed reduced empathic concern and heightened personal distress. The sustained states of stress, anxiety, and fear may exacerbate the development of self-focused (potentially survival) behavior and competitiveness in trauma-exposed individuals as a result of being chronically engaged in subcortically-driven primitive defensive responses (Steuwe et al., 2014, 2015). Individuals who develop under abusive, neglectful, and traumatic circumstances may not develop optimal socio-cognitive processes that in turn mediate altruistic behavior. The lack of altruism demonstrated by patients with PTSD in our study may be attributable to chronic, early childhood abuse and resulting alterations in empathetic functioning.

### Limitations and future directions

There are several limitations to this study, including the small sample size. The generalizability of our results is limited to women who have been exposed to chronic childhood trauma and is not applicable to men, single-blow trauma, or chronic trauma not related to childhood victimization. Education levels were lower in patients than in controls, a common characteristic of studies involving neuropsychiatric populations likely reflecting the influence of illness status on educational attainment. As is also common in studies of trauma-exposed participants, patients had a high burden of illness and co-morbidity; however, symptoms of depression, dissociation, and PTSD severity were not associated with altered performance on the moral reasoning dilemmas presented. Finally, due to our cross-sectional design and a lack of a trauma-exposed control group, we cannot distinguish between the effects of PTSD and exposure to early-life trauma on moral judgment, although interestingly moral reasoning performance was not correlated with current PTSD symptom severity, pointing to longer-term alterations in moral reasoning that may be partially independent of current state. It is probable that these longer-term alterations interact synergistically with current symptom states (e.g., symptoms of shame and guilt) to influence performance.

Critically, the hypothetical high-conflict moral dilemmas used in this study are generally not encountered in real-life; future studies should explore judgment behind moral dilemmas that are more pertinent to real-life situations. Considering that we found differences in moral judgment only when participants were asked to consider their own agency behind the hypothetical actions, future research should ensure morality paradigms maximize perspective-taking in order to create immersive scenarios. Care must be taken to control the types of scenarios delivered to participants in order to avoid presenting trauma-related cues. Unfortunately, reaction time for moral decision-making was not recorded and should be implemented in future studies given that individuals with PTSD due to childhood trauma have shown delayed latencies during identification of prosody (Nazarov, Frewen, et al., 2015) and emotionally salient complex mental states (Nazarov et al., 2014). Other important limitations of the dilemmas include the absence of a rating scale assessing the individual emotional relevance of the dilemmas presented. The social desirability of the choices presented (e.g., to not kill another person) may also affect patterns of responding under conditions requiring an explicit response. In addition, the dilemmas were not constructed to control for potentially different working memory and executive functioning demands across conditions (e.g., physical harm vs. non-physical harm). Future studies may control for these potentially confounding factors and assess further how factors such as individual levels of neuropsychological functioning affect performance.

In our study, we did not use an independent measure of guilt and shame but rather a sub-score from the CAPS; future investigations should utilize more accurate instruments of state and trait measures of guilt and shame. Due to differing definitions, interpretations, quantification, and some research failing to distinguish guilt and shame altogether (Tangney, 1996), the extent to which shame and guilt independently relate to adverse mental health outcomes is difficult to evaluate. Researchers intending to capture the interplay between morality and the experiences of guilt and shame must be cognizant of the distinct underlying psychological constructs of moral emotions (Tangney, 1996; Tangney et al., 2007). Longitudinal studies investigating the interaction of moral judgment and existing feelings of shame and guilt are warranted, particularly concerning treatment interventions targeting symptoms of guilt and shame in PTSD populations. Finally, in light of neuroimaging findings alluding to the role of the default mode network (DMN) and the salience network (SN) in moral reasoning (Chiong et al., 2013) and to disruptions in these networks in PTSD (Daniels, Frewen, McKinnon, & Lanius, 2011), future investigations should longitudinally explore moral reasoning performance before and after DMN-modifying treatments such as electroencephalographic neurofeedback (Kluetsch et al., 2014).

## Conclusions

Here, we demonstrated altered moral judgment processing in women with PTSD related to chronic childhood trauma. Critically, in comparison to healthy women, women with PTSD were less likely to approve utilitarian actions when required to assume their own agency in actions involving the infliction of direct physical harm. The decreased likelihood of utilitarian action approval by the PTSD sample was driven by significantly enhanced endorsement of guilt and shame as consequences of such actions. Within the PTSD sample, endorsement of guilt and shame in moral dilemmas was related to increased severity of current clinical symptoms of guilt. Finally, in dilemmas exploring altruism vs. self-interest, women with PTSD were less likely to morally approve an altruistic action and were less likely to carry out the altruistic action themselves. This study further extends the growing literature on socio-cognitive alterations associated with psychological trauma, the results of which may be applied to interventions aimed at ameliorating impairments in interpersonal functioning, particularly during morally conflicting and emotionally salient social contexts.

## Appendix

### Appendix A—Moral dilemmas administered in this study

#### UTILITARIANISM VS. DEONTOLOGY [PERSONAL]

##### Military Submarine

You are the captain of a military submarine. An onboard explosion has caused you to lose most of your air supply and has injured one of your crewmembers, who is quickly losing blood. The injured crewmember is going to die from his wounds no matter what happens. There isn't enough air for the whole crew. The only way to save the other crewmembers is to shoot dead the injured crewmember so that there will be just enough air for the rest of the crew to survive.

##### Shopping Mall Bomb

You are dealing with a terrorist who is about to set off a bomb in a crowded area. You are holding his teenage son at your headquarters. After lengthy negotiations, you realize that the only way you can stop the terrorist from setting off a bomb that will kill thousands of people, is to break one of his son's arms. You also threaten to break the other arm if he does not give himself up. You also realize that in order to make sure the terrorist believes you, you would have to break the son's limbs yourself and have it videotaped and sent to the boy's father.

##### Boat at Sea

You are on a big boat at sea. There is a fire on the boat and everyone has to evacuate the boat. People get into the lifeboats. All the lifeboats, including yours, have too many people in them. The sea is getting rough, and water is coming in over the sides. If nothing is done the lifeboat will sink and everyone on board will die. A young woman on board proposes to sacrifice herself for the sake of others. If you throw her overboard, the boat will stay afloat and the remaining passengers will be saved. However, she is afraid of the actual drowning, and begs you to cut her throat with your knife and throw her body into the sea.

#### UTILITARIANISM VS. DEONTOLOGY [IMPERSONAL]

##### Vaccine

You work for the government's public health office. Scientists have made a new vaccine to fight a serious disease, and you must decide whether the government will tell people to use it. Almost everyone who uses the vaccine will get protection from this deadly disease. However, a very small number of the people who take the vaccine will get the disease that it is meant to prevent. Still, the experts all agree that the risk of getting the disease is much higher for people who don't take the vaccine than it is for people who do take it.

##### Cancer Drug

You are a scientist at a pharmaceutical company. You have been involved in testing a drug that might cure breast cancer. The drug has been found to be safe for animals, but it is still not clear if it is safe to give it to humans. Your company wants to begin giving this drug to women who are dying of breast cancer. You are worried, however, that the drug might be unsafe for humans and that it could kill some of the women shortly after they take it. On the other hand, there is a strong possibility this drug could cure breast cancer and save many women's lives.

##### Chief of Police

You are the Chief of Police. Your police officers have just caught several people known to be involved in terrorism. The terrorists tell you that they have planted a dangerous bomb in a large shopping center but refuse to tell you which one or in what town it is located. The bomb will explode in a few hours. You estimate that it could cause severe casualties. With the limited amount of time at your disposal, the only way to discover the location of the bomb is to order the police officers to torture the terrorists until they tell them where the bomb is located.

#### COMPASSION VS. SOCIAL ORDER

##### Lying Mayor

You are a writer for your town's newspaper. You have learned that the Mayor is lying about the amount of money the municipality owes to the bank. Many taxpayers in your town are being forced to pay higher taxes because of the Mayor's lies.

##### Doctor's Office

Your sister's boyfriend has just had some blood-work done, and she is interested in finding out the results of these tests. She asked him for the results but he was not very forthcoming. You and your sister begin to wonder whether he is hiding the fact that he has a sexually-transmitted infection. You are working at the medical office where the tests were done, so you could photocopy the results for you and your sister to look at. You know these results are confidential and that you should not violate patient confidentiality. On the other hand, you are truly worried about your sister's health.

##### Employee Fraud

You are the general manager of a big bank. Recently, you have had some cases of fraud by bank employees. In the past, the bank reported all of these cases to the police, and in all cases, the employees were charged with fraud. Your assistant suggests that perhaps some of these employees have had personal problems that had led to the crime. It is in your power to change the policy so that the employees who had personal problems at the time of the crime will not be reported to the police.

##### School Theft

You are a schoolteacher in a rough neighborhood. One of your students is an exceptionally bright and caring individual, who has suffered a lot in life. Although the student has been involved in many crimes in the past, he says he is reformed and plans to attend university. You are tutoring him to prepare him for his entrance exams when you notice that an item has clearly been stolen. When you confront the student, he tells you he deeply regrets taking that item. He has not stolen since he took this item, and vows to never steal again. You are not sure whether you should report the theft to the police. Any further arrests could prevent the student from attending university.

#### ALTRUISM VS. SELF INTEREST

##### Charity

You receive a letter from a highly respected charity. The letter asks you to make a donation of $200. The letter explains that a $200 donation will allow the charity to provide badly needed food and medicine to poor people on the other side of the world. $200 is the amount of money you receive for two days of work at your current job. You consider your financial situation relatively average, but still, it would not be very easy for you to make this kind of donation at this time.

##### Blood Donation

Your good friend's daughter was diagnosed with colon cancer. She needs a specific blood cell donation, and you were found to be a perfect match. The procedure is lengthy and unpleasant, and requires several days of hospitalization. However, it is not painful or dangerous.

### Appendix B—Themes used in qualitative analysis

**Unilateral and Physicalistic**

Unilateral Authority

Status

Rules

Physical Consequences

Labels

**Exchanging and Instrumental**

Preferences

Needs

Freedoms

Exchanges

Equalities

Advantages

**Mutual and Prosocial**

Relationships

Prosocial Intentions

Normative Expectations

Intrapersonal Approval

Generalized Caring

Empathic Role-Taking

**Systemic and Standard**

Standards of Conscience

Societal Requirements

Responsibility

Procedural Equity

Consistent Practices

Character

Basic Rights or Values

**Custom/Additional**

Skepticism

Self-serving

Self-sacrifice

Overwhelmed

Hope/Questioning probability of negative events

Greater Good

Displacing Responsibility

Refusal without justification (“can't do it”)

Alternative Suggestion

## References

[CIT0001] Aguert M, Laval V, Lacroix A, Gil S, Le Bigot L (2013). Inferring emotions from speech prosody: Not so easy at age five. PLoS One.

[CIT0002] Amit E, Greene J.D (2012). You see, the ends don't justify the means: Visual imagery and moral judgment. Psychological Science.

[CIT0003] Beck A.T, Steer R.A, Brown G.K (1996). Manual for the Beck Depression Inventory-II.

[CIT0004] Beer J.S, Heerey E.A, Keltner D, Scabini D, Knight R.T (2003). The regulatory function of self-conscious emotion: Insights from patients with orbitofrontal damage. Journal of Personality and Social Psychology.

[CIT0005] Bernstein D.P, Fink L, Handelsman L, Foote J, Lovejoy M, Wenzel K, Ruggiero J, … (1994). Initial reliability and validity of a new retrospective measure of child abuse and neglect. The American Journal of Psychiatry.

[CIT0006] Berthoz S, Grèzes J, Armony J.L, Passingham R.E, Dolan R.J (2006). Affective response to one's own moral violations. NeuroImage.

[CIT0007] Blair R (2007). The amygdala and ventromedial prefrontal cortex in morality and psychopathy. Trends in Cognitive Sciences.

[CIT0008] Blair R, Sellars C, Strickland I, Clark F, Williams A, Smith M, Jones L (1995). Emotion attributions in the psychopath. Personality and Individual Differences.

[CIT0009] Blake D.D, Weathers F.W, Nagy L.M, Kaloupek D.G, Gusman F.D, Charney D.S, Keane T.M (1995). The development of a clinician-administered PTSD scale. Journal of Traumatic Stress.

[CIT0010] Blakemore S.-J, Choudhury S (2006). Development of the adolescent brain: Implications for executive function and social cognition. Journal of Child Psychology and Psychiatry and Allied Disciplines.

[CIT0011] Bremner J (1999). Acute and chronic responses to psychological trauma: Where do we go from here?. The American Journal of Psychiatry.

[CIT0012] Briere J (2002). MDI, Multiscale Dissociation Inventory: Professional manual.

[CIT0013] Budden A (2009). The role of shame in posttraumatic stress disorder: A proposal for a socio-emotional model for DSM-V. Social Science & Medicine.

[CIT0014] Caruso E.M, Gino F (2011). Blind ethics: Closing one's eyes polarizes moral judgments and discourages dishonest behavior. Cognition.

[CIT0015] Charuvastra A, Cloitre M (2008). Social bonds and posttraumatic stress disorder. Annual Review of Psychology.

[CIT0016] Chiong W, Wilson S.M, D'Esposito M, Kayser A.S, Grossman S.N, Poorzand P, Rankin K.P, … (2013). The salience network causally influences default mode network activity during moral reasoning. Brain: A Journal of Neurology.

[CIT0017] Cima M, Tonnaer F, Hauser M.D (2010). Psychopaths know right from wrong but don't care. Social Cognitive and Affective Neuroscience.

[CIT0018] Cloitre M, Miranda R, Stovall-McClough K.C, Han H (2005). Beyond PTSD: Emotion regulation and interpersonal problems as predictors of functional impairment in survivors of childhood abuse. Behavior Therapy.

[CIT0019] Daniels J.K, Frewen P, McKinnon M.C, Lanius R.A (2011). Default mode alterations in posttraumatic stress disorder related to early-life trauma: A developmental perspective. Journal of Psychiatry & Neuroscience.

[CIT0020] De Hooge I.E, Zeelenberg M, Breugelmans S.M (2007). Moral sentiments and cooperation: Differential influences of shame and guilt. Cognition and Emotion.

[CIT0021] DiLillo D (2001). Interpersonal functioning among women reporting a history of childhood sexual abuse: Empirical findings and methodological issues. Clinical Psychology Review.

[CIT0022] Eisenberg N (2014). Altruistic emotion, cognition, and behavior (PLE: Emotion).

[CIT0023] Eisenberg N, Eggum N.D, Di Giunta L (2010). Empathy-related responding: Associations with prosocial behavior, aggression, and intergroup relations. Social Issues and Policy Review.

[CIT0024] Evans L, McHugh T, Hopwood M, Watt C (2003). Chronic posttraumatic stress disorder and family functioning of Vietnam veterans and their partners. The Australian and New Zealand Journal of Psychiatry.

[CIT0025] FeldmanHall O, Dalgleish T, Evans D, Mobbs D (2015). Empathic concern drives costly altruism. NeuroImage.

[CIT0026] First M.B, Spitzer R.L, Gibbon M, Williams J.B.W (2002). Structured clinical interview for DSM-IV-TR axis I disorders, research version, patient edition.

[CIT0027] Fish-Murray C.C, Koby E, Van der Kolk B, Van der Kolk B.A (1987). Evolving ideas: The effect of abuse on children's thought. Psychological trauma.

[CIT0028] Francati V, Vermetten E, Bremner J.D (2007). Functional neuroimaging studies in posttraumatic stress disorder: Review of current methods and findings. Depression and Anxiety.

[CIT0078] Frewen P. A, Lanius R. A (2006). Toward a psychobiology of posttraumatic self-dysregulation. Annals of the New York Academy of Sciences.

[CIT0029] Frewen P, Lanius R (2015). Healing the traumatized self: Consciousness, neuroscience, treatment.

[CIT0030] Gibbs J.C, Basinger K.S, Fuller D (1992). Moral maturity: Measuring the development of sociomoral reflection.

[CIT0031] Greene J.D, Haidt J (2002). How (and where) does moral judgment work?. Trends in Cognitive Sciences.

[CIT0032] Greene J.D, Morelli S.A, Lowenberg K, Nystrom L.E, Cohen J.D (2008). Cognitive load selectively interferes with utilitarian moral judgment. Cognition.

[CIT0033] Greene J.D, Nystrom L.E, Engell A.D, Darley J.M, Cohen J.D (2004). The neural bases of cognitive conflict and control in moral judgment. Neuron.

[CIT0034] Greene J.D, Sommerville R.B, Nystrom L.E, Darley J.M, Cohen J.D (2001). An fMRI investigation of emotional engagement in moral judgment. Science (New York, N.Y.).

[CIT0035] Haas B.W, Brook M, Remillard L, Ishak A, Anderson I.W, Filkowski M.M (2015). I know how you feel: The warm-altruistic personality profile and the empathic brain. PloS One.

[CIT0036] Herman J.L (1992). Complex PTSD: A syndrome in survivors of prolonged and repeated trauma. Journal of Traumatic Stress.

[CIT0037] Herman J.L, Dearing R.L, Tangney J.P (2011). Shame in the therapy hour.

[CIT0038] Kant I, Beck L.W (1959). Foundations of the metaphysics of morals and what is enlightenment?.

[CIT0039] Kluetsch R.C, Ros T, Théberge J, Frewen P.A, Calhoun V.D, Schmahl C, Lanius R.A, … (2014). Plastic modulation of PTSD resting-state networks and subjective wellbeing by EEG neurofeedback. Acta Psychiatrica Scandinavica.

[CIT0040] Koenigs M, Tranel D (2007). Irrational economic decision-making after ventromedial prefrontal damage: Evidence from the ultimatum game. The Journal of Neuroscience.

[CIT0041] Lanius R.A, Bluhm R, Frewen P.A (2011). How understanding the neurobiology of complex post-traumatic stress disorder can inform clinical practice: A social cognitive and affective neuroscience approach. Acta Psychiatrica Scandinavica.

[CIT0042] Lanius R.A, Bluhm R, Lanius U, Pain C (2006). A review of neuroimaging studies in PTSD: Heterogeneity of response to symptom provocation. Journal of Psychiatric Research.

[CIT0043] Lanius R.A, Brand B, Vermetten E, Frewen P.A, Spiegel D (2012). The dissociative subtype of posttraumatic stress disorder: Rationale, clinical and neurobiological evidence, and implications. Depression and Anxiety.

[CIT0044] Lanius R.A, Frewen P.A, Vermetten E, Yehuda R (2010). Fear conditioning and early life vulnerabilities: Two distinct pathways of emotional dysregulation and brain dysfunction in PTSD. European Journal of Psychotraumatology.

[CIT0045] Lanius R.A, Vermetten E, Loewenstein R.J, Brand B, Schmahl C, Bremner J.D, Spiegel D (2010). Emotion modulation in PTSD: Clinical and neurobiological evidence for a dissociative subtype. The American Journal of Psychiatry.

[CIT0046] Lanius R.A, Williamson P.C, Boksman K, Densmore M, Gupta M, Neufeld R.W.J, Menon R.S, … (2002). Brain activation during script-driven imagery induced dissociative responses in PTSD: A functional magnetic resonance imaging investigation. Biological Psychiatry.

[CIT0047] Leskela J, Dieperink M, Thuras P (2002). Shame and posttraumatic stress disorder. Journal of Traumatic Stress.

[CIT0048] Liberzon I, Sripada C.S (2008). The functional neuroanatomy of PTSD: A critical review. Progress in Brain Research.

[CIT0049] Litz B.T, Stein N, Delaney E, Lebowitz L, Nash W.P, Silva C, Maguen S (2009). Moral injury and moral repair in war veterans: A preliminary model and intervention strategy. Clinical Psychology Review.

[CIT0050] Malti T, Ongley S.F, Killen M, Smetana J (2014). Handbook of moral development.

[CIT0051] Matos M, Pinto-Gouveia J (2010). Shame as a traumatic memory. Clinical Psychology & Psychotherapy.

[CIT0052] Mazza M, Giusti L, Albanese A, Mariano M, Pino M.C, Roncone R (2012). Social cognition disorders in military police officers affected by posttraumatic stress disorder after the attack of An-Nasiriyah in Iraq 2006. Psychiatry Research.

[CIT0053] McKinnon M.C, Moscovitch M (2007). Domain-general contributions to social reasoning: Theory of mind and deontic reasoning re-explored. Cognition.

[CIT0054] Mill J.S, Crisp R (1998). Utilitarianism.

[CIT0055] Moll J, De Oliveira-Souza R (2007a). Moral judgments, emotions and the utilitarian brain. Trends in Cognitive Sciences.

[CIT0056] Moll J, De Oliveira-Souza R (2007b). Response to Greene: Moral sentiments and reason: Friends or foes?. Trends in Cognitive Sciences.

[CIT0057] Moll J, De Oliveira-Souza R, Zahn R (2008). The neural basis of moral cognition: Sentiments, concepts, and values. Annals of the New York Academy of Sciences.

[CIT0058] Moll J, Zahn R, De Oliveira-Souza R, Krueger F, Grafman J (2005). Opinion: The neural basis of human moral cognition. Nature Reviews. Neuroscience.

[CIT0059] Nazarov A, Frewen P, Oremus C, Schellenberg E.G, McKinnon M.C, Lanius R (2015). Comprehension of affective prosody in women with post-traumatic stress disorder related to childhood abuse. Acta Psychiatrica Scandinavica.

[CIT0060] Nazarov A, Frewen P, Parlar M, Oremus C, MacQueen G, McKinnon M, Lanius R (2014). Theory of mind performance in women with posttraumatic stress disorder related to childhood abuse. Acta Psychiatrica Scandinavica.

[CIT0061] Nazarov A, Jetly R, McNeely H, Kiang M, Lanius R, McKinnon M.C (2015). Role of morality in the experience of guilt and shame within the armed forces. Acta Psychiatrica Scandinavica.

[CIT0062] Nietlisbach G, Maercker A, Rössler W, Haker H (2010). Are empathic abilities impaired in posttraumatic stress disorder?. Psychological Reports.

[CIT0063] Northoff G, Bermpohl F (2004). Cortical midline structures and the self. Trends in Cognitive Sciences.

[CIT0064] Parlar M, Frewen P, Nazarov A, Oremus C, MacQueen G, Lanius R, McKinnon M.C (2014). Alterations in empathic responding among women with posttraumatic stress disorder associated with childhood trauma. Brain and Behavior.

[CIT0065] Patil I, Silani G (2014). Reduced empathic concern leads to utilitarian moral judgments in trait alexithymia. Frontiers in Psychology.

[CIT0066] Paxton J.M, Greene J.D (2010). Moral reasoning: Hints and allegations. Topics in Cognitive Science.

[CIT0067] Reniers R.L, Corcoran R, Völlm B.A, Mashru A, Howard R, Liddle P.F (2012). Moral decision-making, ToM, empathy and the default mode network. Biological Psychology.

[CIT0068] Saxe R, Carey S, Kanwisher N (2004). Understanding other minds: Linking developmental psychology and functional neuroimaging. Annual Review of Psychology.

[CIT0069] Steuwe C, Daniels J.K, Frewen P.A, Densmore M, Pannasch S, Beblo T, Lanius R.A, … (2014). Effect of direct eye contact in PTSD related to interpersonal trauma: An fMRI study of activation of an innate alarm system. Social Cognitive and Affective Neuroscience.

[CIT0070] Steuwe C, Daniels J.K, Frewen P.A, Densmore M, Theberge J, Lanius R.A (2015). Effect of direct eye contact in women with PTSD related to interpersonal trauma: Psychophysiological interaction analysis of connectivity of an innate alarm system. Psychiatry Research.

[CIT0071] Tangney J.P (1996). Conceptual and methodological issues in the assessment of shame and guilt. Behaviour Research and Therapy.

[CIT0072] Tangney J.P, Stuewig J, Mashek D.J (2007). Moral emotions and moral behavior. Annual Review of Psychology.

[CIT0073] Thomson J.J (1986). Rights, restitution, and risk: Essays, in moral theory.

[CIT0074] Turiel E, Damon W, Lerner R.M (2008). The development of morality. Child and adolescent development: An advanced course.

[CIT0075] Walker L.J (1989). A longitudinal study of moral reasoning. Child Development.

[CIT0076] Wellman H.M, Cross D, Watson J (2001). Meta-analysis of theory-of-mind development: The truth about false belief. Child Development.

[CIT0077] Yang M.L, Yang C.C, Chiou W.B (2010). When guilt leads to other orientation and shame leads to egocentric self-focus: Effects of differential priming of negative affects on perspective taking. Social Behavior and Personality: An International Journal.

